# The association between cannabis use and suicidal behavior in patients with psychiatric disorders: an analysis of sex differences

**DOI:** 10.1186/s13293-018-0182-x

**Published:** 2018-06-11

**Authors:** Leen Naji, Tea Rosic, Brittany Dennis, Meha Bhatt, Nitika Sanger, Jackie Hudson, Natalia Mouravska, Lehana Thabane, Zainab Samaan

**Affiliations:** 1 Department of Family Medicine, McMaster University, Hamilton, Canada; 2 Department of Psychiatry and Behavioral Neurosciences, McMaster University, 100 West 5th Street, Mood Disorders Program, Hamilton, Ontario L8N 3K7 Canada; 3 St. George’s University of London, London, UK; 4 Department of Health Research Methods, Evidence, and Impact, McMaster University, Hamilton, Canada; 5 Medical Science Graduate Program, McMaster University, Hamilton, Canada; 6 Hamilton Health Sciences, Hamilton, Ontario Canada; 7 Departments of Pediatrics and Anesthesia, McMaster University, Hamilton, Canada; 8 Biostatistics Unit, Father Sean O’Sullivan Research Centre, St Joseph’s Healthcare, Hamilton, Canada; 9 Population Genomic Program, Chanchalani Research Centre, McMaster University, Hamilton, Canada

**Keywords:** Cannabis, Suicide, Sex differences, Psychiatric disorder

## Abstract

**Background:**

Cannabis is the most commonly used illicit drug. In the general population, its use has been linked to a heightened propensity for suicidal behavior (SB). We hypothesize that this association varies in patients with psychiatric disorders. SB is known to vary by sex and therefore an investigation of cannabis’ association with SB must consider sex differences. The purpose of this study is to investigate the association between cannabis use and suicide attempts in men and women with psychiatric disorders.

**Methods:**

We merged data collected for two studies based in Ontario, Canada (*n* = 985). We employed a multivariable logistic regression to assess the association between cannabis use and suicide attempts in men and women with psychiatric disorders.

**Results:**

We analyzed data from 465 men and 444 women. Amongst these, 112 men and 158 women had attempted suicide. The average age of our participants was 40 years (standard deviation (SD) 12.4). We found no significant association between suicide attempts and cannabis use in men (odds ratio (OR) = 1.34, 95% confidence interval (CI) 0.81, 2.22, *p* = 0.260) or women (OR = 0.97, 95% CI 0.61, 1.54, *p* = 0.884). In a sensitivity analysis using a sample of patients with substance use disorder only, the heaviness of cannabis use was associated with small but significant association with SB in men (OR = 1.03, 95% CI 1.01, 1.05, *p* = 0.007).

**Conclusion:**

Our findings indicate that there is no association between cannabis use and suicidal behavior in men or women with psychiatric disorders unlike what was reported for the general population, though the heaviness of cannabis use may have an effect in men. The impact of cannabis use in psychiatric disorders needs ongoing examination in light of its common use, impending legalization with expected increased access and the uncertainty about cannabis’ effects on prognosis of psychiatric disorders. In addition, research should continue to investigate modifiable risk factors of SB in this population of which cannabis is not a significant factor based on this study.

## Background

Cannabis is the most commonly used illicit substance worldwide, and its consumption is only expected to increase as more jurisdictions, including Canada, legalize recreational use [1–4]. While this legislation may serve to lower societal costs associated with criminal penalties, the increased consumption of cannabis will likely lead to an increased prevalence of its various detrimental consequences on memory, judgment, cognition, and mental health [3–5].

Cannabis use has consistently been shown to be associated with a heightened propensity for suicidal behavior (SB) in the general population [6–9]. A twin study, for instance, revealed that amongst a sample of 277 same-sex twin pairs, cannabis users were 2.9 times more likely to attempt suicide than their non-cannabis-dependent co-twin.[6]. A prospective cohort study also found a robust association between regular cannabis use and suicide attempt after adjusting for important cofounders (odds ratio (OR) = 2.9, 95% confidence interval (CI) 1.3, 6.1) [9].

However, the majority of the studies investigating the aforementioned association included general population cohorts and limited characterization of psychopathology in the participants being studied. Given the high prevalence of cannabis use in patients with psychiatric disorders and the detrimental impact of suicide on individuals and societies, uncovering the associations, if any, between cannabis use and risk of suicide in this patient population is critical [10–12].

Furthermore, there is an abundance of evidence to suggest that women are more frequently diagnosed with psychiatric illnesses, in particular anxiety and depression, compared to men [13–15]. Women have also repeatedly been shown to have an increased risk of attempting suicide in the general population [16, 17]. Given this, we sought to determine the association between cannabis use and suicide attempt in women and men with psychiatric disorders separately. We hypothesize that cannabis use will be associated with an even greater risk of attempting suicide in women compared to men, given that women have been shown to be more susceptible to the detrimental mental, medical and social consequences of substance use including cannabis [15, 18–21]. For instance, compared to men, women have repeatedly been shown to become more rapidly addicted to drugs, suffer from more severe medical problems despite less heavy use (e.g., chronic obstructive pulmonary disease or alcoholic liver disease), and report more intense subjective responses to drug use [15, 22, 23]. A study by Patton et al. found that although the prevalence and heaviness of cannabis use is greater amongst men, women who used cannabis daily had more than a fivefold increase in the risk of having depression or anxiety compared to non-users, whereas no association was found in the same analysis of men [24].

This is the first study, to our knowledge, to investigate the association between cannabis use and suicidal behavior in the psychiatric population by sex. Our findings will assist in stratifying the risk of suicide attempt in psychiatric patients, who are amongst the most likely to attempt suicide [12, 19]. Provided the World Health Organization’s (WHO) Mental Health Action Plan to reduce the rate of suicide by 10% by 2020, as well as the legalization of cannabis in Canada to start September 2018, our study is both timely and relevant [18].

## Methods

### Data collection

**Fig. 1:**
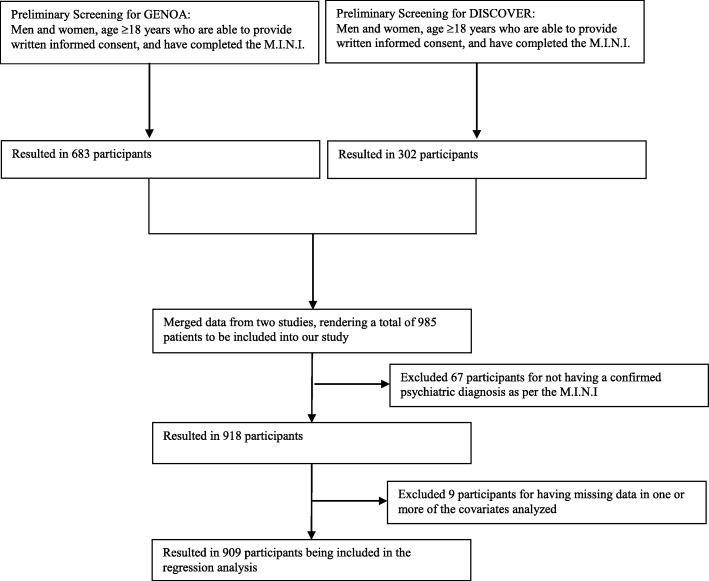
Participant inclusion diagram

We merged patient data that were obtained using similar protocols to collect homogenous data on adults with psychiatric disorders for two studies whose methods have previously been described [20, 25]. Briefly, these two studies are the Genetics of Opioid Addiction (GENOA) study, a prospective cohort study of opioid use disorder using structured scales to assign psychiatric diagnoses, and the Determinants of Suicidal Behavior: Conventional and Emergent Risk (DISCOVER) study, a case-control study of SB using the same diagnostic methods to reach a psychiatric diagnosis including substance use [20, 25]. These studies were approved by the Hamilton Integrated Research Ethics Board (10-661, 11-3479, and 11-056). In order to be included into the current study, participants had to be 18 years of age or older, provided written informed consent, must have been interviewed by trained research personnel using the Mini-International Neuropsychiatric Interview (M.I.N.I.) and must have also had at least one confirmed psychiatric diagnosis based on the M.I.N.I. The M.I.N.I. is a fully validated alternative to the Structured Clinical Interview for the Diagnostic and Statistical Manual of Mental Disorders’ (DSM-IV) diagnoses and the Composite International Diagnostic Interview for the International Classification of Diseases, Tenth Revision (ICD-10) [26]. From both studies, 683 and 302 patients were screened for inclusion into our present study (*n* = 985). See participants’ flow diagram (Fig. 1).

Our primary outcome was the presence or absence of a lifetime suicide attempt based on patients’ dichotomous response to the question “Did you ever make a suicide attempt?” on the M.I.N.I., coded “yes” or “no.” Cannabis use was also a dichotomous covariate based on participants’ response to the question on the M.I.N.I., “In the past 12 months, did you take any of [hashish, “hash”, THC, “pot”, “grass”, “weed”, or “reefer”] more than once, to get high, to feel elated, to get “a buzz” or to change your mood?”. The past 12 months of cannabis use is commonly used in published literature to identify cannabis users [9, 27]. Data on heaviness of cannabis use in the GENOA sample was also obtained through participants’ reported “average number of days of cannabis use in the past 30 days” as part of the Maudsley Addiction Profile [28]. Demographic data including age, sex, marital and employment status were obtained through face-to-face interviews with all study participants as previously described [20, 25]. Psychiatric diagnoses were made if patients met the criteria on the M.I.N.I. for that specific diagnosis during their intake interview. Patients diagnosed with co-occurring psychiatric disorders were classified as having a single principal diagnosis as per the DSM-IV hierarchy of diagnoses [29].

### Statistical analysis

We used descriptive statistics to summarize the participants’ demographic and baseline characteristics. We reported continuous variables using mean (standard deviation (SD)) and categorical variables using proportions (percent).

A multi-variable logistic regression model with suicide attempt as the dependent variable and cannabis use as the explanatory variable was employed to determine the strength of the association between cannabis use and suicide attempts in the entire sample, while adjusting for the following clinically relevant factors: sex, age, marital status, employment status, and psychiatric diagnoses. Two additional multi-variable logistic regressions using the same independent and dependent variables described above were conducted for men and women separately. Lastly, a separate analysis was conducted including the interaction variable “sex*cannabis use.” The most commonly found primary psychiatric diagnoses were substance use disorders (including drugs other than cannabis), mood disorders (including depression and bipolar disorders), anxiety disorders (including generalized anxiety, social anxiety, obsessive-compulsive and post-traumatic stress disorders), and psychotic disorders (including schizophrenia, schizophreniform, schizoaffective, delusional, and brief psychotic disorder). However, the covariate “substance use disorder” (which included drugs other than cannabis) was eliminated from the analysis due to a high level of collinearity as per the variance inflation factor.

We conducted a sensitivity analysis using data from the GENOA sample to look at the impact, if any, of heaviness of cannabis use on suicide attempt in men and women with psychiatric diagnoses. Lastly, we conducted secondary analyses investigating the association between cannabis use (yes/no) and suicide attempts amongst GENOA and DISCOVER participants separately, to ensure robustness of our findings. Our study was adequately powered to detect an association using our logistic regression analyses with well more than 10 events per predictor variable [30]. Specifically, we analyzed data from 465 men and 444 women against 7 covariates (age, employment, marital status, cannabis use, mood, anxiety, and psychotic disorders). All statistical analyses were conducted using Stata 13 [31].

## Results

### Participant characteristics

Amongst the 985 participants included in our study, 76 participants were excluded for having missing data in one or more of the covariates analyzed (Fig. 1). Therefore, 909 participants were included in the regression analysis.

The mean age of participants included in our study was 40.2 years (SD = 12.4). Amongst the 909 participants, more women (*n* = 158) than men (*n* = 112) reported attempting suicide (OR = 1.48, 95% CI 1.09, 2.01, *p* = 0.012). Men reported twice as much rate of cannabis use compared to women (45.6 versus 23.4%, respectively). Please see Table 1 for a full description of the participants' characteristics.

**Table 1:** Participant characteristics (*n* = 909)

Participant characteristic		Total (*n* = 909)	Men (*n* = 465)	Women (*n* = 444)
		Mean (SD)	Mean (SD)	Mean (SD)
Current age (years)		40.2 (12.4)	40.7 (12.3)	39.7 (12.5)
		*N* (% of total)	*N* (% of men)	*N* (% of women)
History of suicide attempt	No	639 (70.3)	353 (75.9)	286 (64.4)
	Yes	270 (29.7)	112 (24.1)	158 (35.6)
Employed	No	598 (65.8)	281 (60.4)	317 (71.4)
	Yes	311 (34.2)	184 (39.6)	127 (28.6)
Marital status	Married or living with partner	277 (30.5)	139 (29.9)	138 (31.1)
	Other	632 (69.5)	326 (70.1)	306 (68.9)
Cannabis use	No	571 (62.8)	253 (54.4)	318 (71.6)
	Yes	338 (37.2)	212 (45.6)	126 (23.4)
Substance use disorder	No	172 (18.9)	64 (13.8)	108 (23.3)
	Yes	737 (81.1)	401 (86.2)	336 (75.7)
Psychotic disorder	No	898 (98.8)	459 (98.7)	439 (98.9)
	Yes	11 (1.21)	6 (1.29)	5 (1.13)
Mood disorder	No	758 (83.4)	409 (88.0)	349 (78.6)
	Yes	151 (16.6)	56 (12.0)	95 (21.4)
Anxiety disorder	No	899 (98.9)	463 (99.6)	436 (98.2)
	Yes	10 (1.10)	2 (0.43)	8 (1.80)

### Suicide attempts and cannabis use

No significant association was found between cannabis use and suicide attempts amongst the total sample of psychiatric patients (OR = 1.08, 95% CI 0.77, 1.51, *p* = 0.663). However, our findings did reveal that amongst participants with psychiatric disorders, having a mood disorder (OR = 2.83, 95% CI 1.90, 4.23, *p* < 0.001) or being a woman (OR = 1.48, 95% CI 1.09, 2.01, *p* = 0.012) is associated with an increased risk of suicide attempt, while having a job is protective against suicide attempts (OR = 0.34, 95% CI 0.24, 0.48, *p* < 0.001). When the same statistical model described above is used to analyze GENOA and DISCOVER data separately, the association between cannabis use and suicide attempt remained insignificant (data not shown). Lastly, the interaction term “sex*cannabis use” remained insignificant when included in the logistic regression model (data not shown).

### Sex differences

Cannabis use was not found to be significantly associated with suicide attempts in women (OR = 0.97, 95% CI 0.61, 1.54, *p* = 0.884) or men (OR = 1.34, 95% CI 0.81, 2.22, *p* = 0.260). Please see Table 2.

**Table 2:** Association between cannabis use (yes/no) and suicide attempt in psychiatric patients

	Women (*n* = 444)		Men (*n* = 465)	
Covariates	Odds ratio (95% CI)	*p* value	Odds ratio (95% CI)	*p* value
Marital status	1.03 (0.67, 1.61)	0.880	0.62 (0.36, 1.07)	0.084
Employed	0.41 (0.25, 0.67)	< 0.001	0.26 (0.15, 0.44)	< 0.001
Age	0.99 (0.98, 1.01)	0.406	1.00 (0.99, 1.02)	0.646
Cannabis use	0.97 (0.61, 1.54)	0.884	1.34 (0.81, 2.22)	0.260
Psychotic disorder	3.34 (0.52, 21.4)	0.204	3.50 (0.65, 18.9)	0.146
Anxiety disorder	1.41 (0.32, 6.25)	0.651	–	–
Mood disorder	2.22 (1.33, 3.71)	0.002	4.57 (2.36, 8.87)	< 0.001

### Sensitivity analysis: heaviness of cannabis use

Within the GENOA sample, heaviness of cannabis use was found to be significantly associated with suicide attempt in men (OR = 1.03, 95% CI 1.01, 1.05, *p* = 0.007), but not women (OR = 0.99, 95% CI 0.97, 1.01, *p* = 0.277). See Table 3 in Appendix.

## Discussion

Findings from this study suggest that amongst patients with a psychiatric comorbidity, women, unemployed individuals, and those with mood disorders are at a significantly heightened risk of attempting suicide. Heaviness of cannabis use was also found to be associated with an increased risk of suicide attempt in men in a subset of the study sample. Contrastingly, no association was found between the risk of attempting suicide and cannabis use, marital status, age, or having an anxiety or psychotic disorder. Interestingly, the negative impact of a mood disorder and the protective effect of employment were more pronounced in men compared to women with psychiatric disorders.

While cannabis use in the general population leads to an increased risk of suicide attempts as seen in some studies, our findings suggest that this association does not hold true in a large cohort of psychiatric patients who are at an already heightened risk of attempting suicide [6–9]. Heaviness of cannabis use was, however, found to have a slight but significant association with suicide attempt in men in a subset of our study sample. The pathophysiological link between cannabis use and suicidal behavior in the general population has yet to be established, though both direct and indirect associations between cannabis use and suicide attempt have been hypothesized [9]. One hypothesis suggests that tetrahydrocannabinol (THC, the active ingredient of cannabis) has direct neurophysiological effects that lead to impaired cognition and behavior, whereas another hypothesis suggests that cannabis use indirectly increases one’s risk for suicide attempt because cannabis users tend to have other predisposing social factors that increase their risk of attempting suicide [9]. Having a psychiatric disorder including other substance use is likely associated with altered neurophysiological state and therefore the effect of cannabis is less well known and difficult to isolate from the underlying psychopathology [32, 33].

It is also likely that the association between cannabis use and suicide risk described in the literature varies by the type of suicidal behavior. We have previously shown that certain epidemiological observations of suicide risk factors, such as obesity, differ for suicide ideation, attempts, and completed suicide [34]. In addition, we have also found that genetic risk factors associated with suicide also varied by the type of suicidal behavior [35]. Taken together, these observations call for homogenous definitions of suicidal behaviors in order to infer conclusions with any certainty on the association between certain risk factors and suicide.

Our significant findings related to suicide attempts and risk factors are mostly supported by existing literature. Previous studies have established that while men are at an increased risk of completing suicide, the prevalence of attempted suicide is significantly higher in women amongst the general population [16, 17]. Amongst psychiatric disorders, mood disorders are the most strongly associated with suicidal attempts [36]. Similarly, unemployment has been linked to a heightened risk of suicide. Whether this association is directly causal or indirectly associated with suicide by precipitating other risk factors for suicide, such as depression, has yet to be established [17, 37, 38]. Interestingly, the association between having a mood disorder and attempting suicide was twice as prominent in men as it was in women. Similarly, employment was more protective against suicide attempt in men compared to women. While such differences have not been previously reported, a plausible explanation may be the fact that women are already at a heightened risk of attempting suicide such that these additional predisposing factors may play a smaller role. A similar rationale may explain the significant association between heaviness of cannabis use and suicide attempt in men but not in women. This finding has not previously been reported in this population, and previous literature has actually revealed that even amongst cannabis users, women are more likely than men to attempt suicide [9]. It is possible that, given that women experience deleterious effects of cannabis use at lower doses and more frequently than men as we describe above, the dose response relationship is less pronounced [22, 24]. Ultimately, our study adds to the existing body of literature showing that the elevated suicidal behavior risk amongst women, the unemployed, and those with a mood disorder seen in the general population remains consistent in a cohort of psychiatric patients and that the effects of unemployment, mood disorders, and heavy cannabis use are more pronounced in men compared to women.

Our current study findings advance knowledge regarding suicide risk in psychiatric patients and warrant further investigation into the effect of cannabis on specific psychiatric disorders. With the WHO’s plans to reduce suicide rate by 10% by 2020, it is imperative that we establish clear risk factors to assist in stratifying individuals’ suicidal risk [18]. While such data exist on a general population level, they are lacking amongst psychiatric patients who comprise a large proportion of patients with suicidal behavior. The prevalence of suicide attempt in our patient sample (29.7%) falls on the higher end of the spectrum of what has previously been reported for psychiatric patients [39]. Given that 50% of patients who attempt suicide are known to have a concurrent substance use disorder, this finding is expected as the majority of patients in our sample have a substance use disorder [40].

Our study reveals that in a large population of psychiatric patients, women, those who are unemployed, those who have a mood disorder, and men with heavier cannabis use are at a heightened risk of attempting suicide and may therefore require closer follow-up, additional counseling, and/or screening for underlying mental health processes to mitigate the risk of SB [41]. Contrastingly, cannabis use as a whole does not seem to add to the suicide risk in this patient population despite its established association with suicide risk in the general population, though this effect may vary in certain subgroups and based on the amounts used. Nonetheless, cannabis use has been associated with other psychopathology such as mood and psychotic symptoms which may in turn increase the risk of suicide and therefore cannabis use in this high-risk population should not be overlooked [42].

### Limitations

It is important to acknowledge the limitations of our findings. Firstly, we merged data from two studies that recruited patients for different purposes. However, the outcomes and covariates we analyzed in the present study were collected from all patients using the same consistent case report forms and the M.I.N.I., meaning that it should not have had an impact on our findings. Additionally, all patients included met the inclusion criteria of our present study, including the presence of a psychiatric diagnosis. It is also worth reiterating that our findings remained unchanged when data from both studies were analyzed separately, indicating robustness of our findings. However, when data on heaviness of cannabis use were investigated, which were available from one study only (GENOA), we noticed a significant association between heaviness of cannabis use and SB in men. This may indicate that the amount and/or frequency of cannabis use may have negative effects on SB and that further studies should examine the amount and frequency of cannabis use in psychiatric patients if we are to draw any firm conclusions.

Secondly, cannabis use and suicide attempt were based on self-report, inevitably subjecting our findings to social desirability bias. However, we can argue that the self-reported use of cannabis is relatively accurate despite the expected bias. In a previous study, we have shown self-reported cannabis use to be significantly associated with urine drug screen for cannabis with 79.9% sensitivity and 80.0% specificity [43]. Our primary question assessing cannabis use also specifies recreational use, which may exclude participants who believe their illicitly obtained cannabis is for medical use or those who obtain medicinal cannabis but use it recreationally, thus potentially biasing our findings towards the null. Once again, this is unlikely to have a major impact on the findings given that approximately 90% of adult cannabis users report recreational use [44].

Moreover, while it is important to identify suicide attempts in a longitudinal study, this is challenging due to the low incidence of suicide attempts and the need for a very large cohort that must be followed for a lengthy period of time making the study not feasible and the cost prohibitive. As such, the majority of the literature on cannabis use and suicide attempt relies on self-report in cross-sectional studies [6, 9]. It is also worth noting that we wanted to identify if differences exist based on how we captured the suicide question and thus conducted a secondary analysis using data from the 231 participants for whom we had access to hospital records of clinically documented suicide attempt, and we found that the association between cannabis use and suicide attempt (as per hospital records) remained insignificant (data not shown).

Lastly, given that our sample comprised of a large number of patients with opioid use disorder, it is possible that the established association between opioid use and SB as well as unintentional overdoses may have confounded our findings [45–47]. However, it is unlikely to have changed our results given that our results remained unchanged in a subgroup analysis of DISCOVER patients of whom a minority were patients with substance use disorders. Nonetheless, it may be worthwhile for future studies investigating the association between cannabis use and suicide attempt in psychiatric patients to stratify findings based on specific psychiatric comorbidities.

## Conclusion

While previous research has demonstrated a positive association between cannabis use and suicide attempt in the general population [6–8], our study reveals that such association does not exist amongst both men and women with psychiatric disorders. However, the heaviness of cannabis use in men had a modest but significant association with suicide attempts in a subset of this study. The impact of cannabis use, although common, in people with psychiatric disorders is therefore different than that in the general population. This study identified significant risk factors for suicide attempts in psychiatric patients including being a women, being unemployed, and having a mood disorder, in keeping with previous research [16, 37, 38]. Findings from this study may serve to educate health professionals when stratifying patients’ risk of suicide as well as policy makers when reviewing current strategies to mitigate suicide risk. This may include more aggressive treatment of mood disorders in primary care, more social services to support patients with psychiatric disorders to assist in job placements, skills development, and programs to encourage return to work plans. Further data on cannabis use including heaviness are also needed to identify those at risk of suicide attempts. Taken together, our findings provide data upon which we may base decisions when striving towards achieving the WHO’s Mental Health Action Plan to reduce the rate of suicide by 10% by 2020, by addressing modifiable risk factors such as unemployment as described above [18].

## Appendix

**Table 3:** Association between *heaviness* of cannabis use and suicide attempt in psychiatric patients

	Women (*n* = 314)		Men (*n* = 364)	
Covariates	Odds ratio (95% CI)	*p* value	Odds ratio (95% CI)	*p* value
Marital status	1.01 (0.57, 1.79)	0.974	0.65 (0.32, 1.31)	0.226
Employed	0.33 (0.17, 0.66)	0.002	0.24 (0.11, 0.50)	< 0.001
Age	0.98 (0.96, 1.01)	0.185	1.01 (0.98, 1.04)	0.566
Cannabis days used	0.99 (0.97, 1.01)	0.277	1.03 (1.01, 1.05)	0.007
Psychotic disorder	2.91 (0.76, 11.1)	0.120	1.44 (0.27, 7.65)	0.669
Anxiety disorder	2.21 (0.94, 5.20)	0.068	3.27 (1.42, 7.57)	0.006
Mood disorder	3.75 (1.85, 7.60)	< 0.001	3.46 (1.75, 6.85)	< 0.000

This table contains the results of the sensitivity analysis investigating the association between the heaviness of cannabis use and suicide attempts amongst a sample of patients with substance use disorders. Heaviness of cannabis use was found to have a modest but significant association with suicide attempt in men (OR = 1.03, 95% CI 1.01, 1.05, *p* = 0.007), but not women (OR = 0.99, 95% CI 0.97, 1.01, *p* = 0.277)

## Abbreviations

CI: Confidence interval
DISCOVER: Determinants of Suicidal Behavior: Conventional and Emergent Risk
DSM-IV: Diagnostic and Statistical Manual of Mental Disorders, Fourth Edition
GENOA: Genetics of Opioid Addiction
ICD-10: International Classification of Diseases, Tenth Revision
M.I.N.I.: Mini-International Neuropsychiatric Interview
OR: Odds ratio
SB: Suicidal behavior
SD: Standard deviation
THC: Tetrahydrocannabinol
WHO: World Health Organization
